# The Amyloid Cascade Hypothesis in Alzheimer’s Disease: Should We Change Our Thinking?

**DOI:** 10.3390/biom13030453

**Published:** 2023-03-01

**Authors:** Markku Kurkinen, Michał Fułek, Katarzyna Fułek, Jan Aleksander Beszłej, Donata Kurpas, Jerzy Leszek

**Affiliations:** 1 Biomed Industries, Inc., San Jose, CA 95131, USA; 2 Department and Clinic of Internal Medicine, Occupational Diseases, Hypertension and Clinical Oncology, Wroclaw Medical University, 50-556 Wroclaw, Poland; 3 Department and Clinic of Otolaryngology, Head and Neck Surgery, Wroclaw Medical University, 50-556 Wroclaw, Poland; 4 Department and Clinic of Psychiatry, Wroclaw Medical University, 50-367 Wroclaw, Poland; 5 Department of Family Medicine, Wroclaw Medical University, 51-141 Wroclaw, Poland

**Keywords:** amyloid hypothesis, astrocyte, dementia, EAAT2, E280A, presenilin

## Abstract

Old age increases the risk of Alzheimer’s disease (AD), the most common neurodegenerative disease, a devastating disorder of the human mind and the leading cause of dementia. Worldwide, 50 million people have the disease, and it is estimated that there will be 150 million by 2050. Today, healthcare for AD patients consumes 1% of the global economy. According to the amyloid cascade hypothesis, AD begins in the brain by accumulating and aggregating Aβ peptides and forming β-amyloid fibrils (Aβ42). However, in clinical trials, reducing Aβ peptide production and amyloid formation in the brain did not slow cognitive decline or improve daily life in AD patients. Prevention studies in cognitively unimpaired people at high risk or genetically destined to develop AD also have not slowed cognitive decline. These observations argue against the amyloid hypothesis of AD etiology, its development, and disease mechanisms. Here, we look at other avenues in the research of AD, such as the presenilin hypothesis, synaptic glutamate signaling, and the role of astrocytes and the glutamate transporter EAAT2 in the development of AD.

## 1. Introduction

Old age comes with many geriatric syndromes, disabilities, and diseases [1]. Yet, nothing compares to Alzheimer’s disease (AD), a most devastating disorder of the human mind and the major cause of dementia. Alois Alzheimer called it “eine eigenartige Erkrankung der Hirnrinde” (a peculiar disease of the cortex) [2].

AD is the most common aging-associated neurodegenerative disease, diagnosed by slowly progressive and irreversible memory loss and other disturbance in cognition, followed by remarkable changes in behavior and personality, and, in the end, loss of self. The last-mentioned term “Loss of self” means that subjective but realistic self-experiencing in dementia is gradually degrading. Some researchers such as Bomilcar et al. describe this process in a multidimensional way [3]. AD is characterized by extracellular amyloid plaques and intracellular neurofibrillary tangles in the brain. Advanced or old age and family history of dementia are the only high-risk factors of AD. These are the risks we cannot do anything about. Other risks include cerebrovascular and cardiovascular diseases, diabetes, head trauma, obesity, psychiatric diseases, and stroke [4,5,6]. APOE4 is the only major genetic risk factor of AD [7]. Inherited dominant mutations in the APP, PS1 or PS2 genes cause 1% of AD, the early-onset familial forms of AD [8,9,10]. APOE e4 increases risk for AD and is also associated with an earlier age of disease onset. Having one or two APOE e4 alleles increases the risk of developing AD, even very early in life in the absence of most AD pathological changes in the brain. About 25 percent of people carry one copy of APOE e4, and 2 to 3 percent carry two copies. Approximately 15% to 25% of the general population carries an APOE e4 allele; APOE e3—the most common—does not seem to affect the risk of AD. About 2 to 3 percent of the world’s population have two copies of APOE e4. Studies show that up to 60% of them will develop AD by age 85, compared with 10 to 15 percent of the general population [11].

AD is diagnosed every 3 s, with the incidence of 10% in people at age 65, 20% at 75, and 40% at 85. Worldwide, 50 million people have AD, estimated to reach 150 million in 2050. In the US, $1 billion a day is spent for healthcare of 6.2 million people living with AD at home or long-term care facilities [12,13,14]. In 2020, the National Institute of Aging supported AD research and clinical trial studies with $2.8 billion [15].

## 2. APP and Aβ Peptides

APP (amyloid precursor protein), a membrane protein with one transmembrane domain, is so named after its proteolytic metabolism, which generates Aβ peptides forming amyloid, an insoluble extracellular aggregate of β-sheet fibrils. Proteinase β-secretase cuts APP outside the membrane, followed by γ-secretase which cuts APP in the middle of the transmembrane domain, thereby releasing 37–43 amino acid-long Aβ peptides, most often the Aβ40, and then the Aβ42 peptide. α-secretase, which cuts APP outside the membrane in the Aβ domain, prevents Aβ peptide production. γ-secretase is made of four proteins, with presenilin PS1 or PS2 as the proteolytic enzyme component. Aβ peptides are generated inside the cell on endosomal membranes [16,17,18,19,20]. APP is expressed in most tissues and cells, including erythrocytes, leucocytes, and platelets [21,22,23,24]. In the blood, platelets have the most APP and Aβ peptides.

APP was cloned by four groups in 1987, and the first APP mutation causing early-onset familial forms of AD was reported in 1987. John Hardy has written an interesting memoir of those days [25]. Cloned from a brain cDNA library as a 675-amino acid protein by Müller-Hill and colleagues, APP has “features characteristic of glycosylated cell-surface receptors” [26].

## 3. The Amyloid Cascade Hypothesis

”While there may be many causes of Alzheimer’s disease (AD), the same pathological sequence of events […] is likely to occur in all cases. […] The pathological cascade for the disease process is likely to be beta-amyloid deposition—tau phosphorylation and tangle formation—neuronal death. The development of biochemical understanding of this pathological cascade will facilitate the rational design of drugs to intervene in this process.” Thus wrote John Hardy and David Allsop in 1991 [27].

According to the amyloid cascade hypothesis [25,28,29], AD begins in the brain with Aβ peptide accumulation, aggregation, and amyloid formation. Formulated in 1991-1992, the hypothesis has dominated AD research, drug discovery, and clinical trial studies ever since [30]. The hypothesis is supported by the molecular genetics of the early-onset familial forms of AD, caused by inherited dominant mutations in the APP, PS1 or PS2 genes. Some 300 pathogenic mutations have been identified which cause AD at age 22–60, the age of onset depending on the gene and the particular mutation. For example, the PS1 mutation E280A causes AD at the median age of 49. PS1 has the most mutations, which can result in increased, decreased or no Aβ peptide production, or in increased or decreased Aβ42/40 ratio (see below), Aβ42 being the more hydrophobic peptide prone to aggregation and amyloid formation [31,32,33,34].

Several observations and experimental studies are against the hypothesis. The natural history of AD progression does not correlate with brain amyloid formation, therefore amyloid cannot be causally linked to AD [35,36]. Brain amyloid PET scans of cognitively unimpaired people often look the same as the PET scans of people with AD [37]. At brain autopsy, 30% of people without AD had typical amyloid formation characteristics of AD brains [38,39]. In short, as David Snowdon put it in his great ‘Nun Study’ in 1997: “Brain amyloid is not synonymous with dementia” [40].

The amyloid cascade hypothesis in Alzheimer’s disease is also not supported by the ambiguous, quite controversial results of clinical trials of drugs with β- or γ-secretase inhibitors or with anti-Aβ antibodies, especially clinical trials before the introduction of aducanumab to treatment. Two identical Phase 3 clinical trials, EMERGE and ENGAGE, evaluated the safety and efficacy of aducanumab in the treatment of Alzheimer’s disease. The data analysis conclusions at the end of the study at 78 weeks were as follows: In EMERGE, high-dose aducanumab reduced the severity of clinical dementia as measured by dementia severity scales. In the ENGAGE study, aducanumab did not reduce pre-study clinical worsening. Even when the drugs reduced the production of Aβ peptides and amyloid in the brain, they did not clearly slow cognitive decline, or slowed it only slightly. Worse still, the drugs often harmed the study volunteers, causing serious health problems including infections, skin cancers, cerebral vascular edema, cerebral microhemorrhages, severe cognitive decline, and death [41,42].

## 4. AD Biomarkers

Development of aging-associated diseases and disabilities happens over many decades, before the first signs and symptoms appear, such as high blood glucose level in diabetes and high cholesterol level and elevated blood pressure in cerebrovascular and cardiovascular diseases [43]. Glucose- and cholesterol-lowering drugs, blood pressure drugs, as well as simple lifestyle changes offer effective prevention, treatment, and disease management. AD has no signs and symptoms until it is diagnosed as such, when there is not much that can be done to help and treat the patient [44]. Current AD drug therapies with acetylcholine esterase inhibitors (donepezil, galantamine, and rivastigmine), or memantine, an inhibitor of NMDA receptor and synaptic glutamate signaling, only provide symptomatic and temporary relief; they do not stop or slow the course of AD progression [45,46]. Hence, research efforts to find biomarkers forecasting AD before it begins are good news for everybody [47,48,49]. Sensitive and specific AD biomarkers could help in early diagnosis, prognosis, and counseling in prevention and treatment of AD with disease-modifying therapies (when they become available).

In recent years, a conceptual shift occurred in the field of Alzheimer’s disease considering the disease as a continuum from preclinical disease characterized by normal cognition and abnormal brain biomarkers to mild cognitive impairment and then clinically apparent dementia. Neuroimaging and CSF biomarkers are being used to detect preclinical AD. There is increasing awareness of the appearance of extracellular amyloid B (Abeta) plaques and neurofibrillary tangles in the intra-cellular environment, the best-known hallmarks of AD, but they are not the ultimate cause of AD. Especially alterations in the blood-brain barrier have also emerged as early markers of this disease. Neuropathology of AD begins many years before symptoms appear and as much as 30 years before dementia occurs. New tests might be able to diagnose the disease when symptoms are very mild or even before the symptoms start. It is very important taking into account that early intervention could offer the best chance of therapeutic success in the future. However, in the absence of disease-modifying therapies, AD biomarkers face an ethical issue in medical practice: forecasting AD decades before its clinical onset may do more harm than good [50,51,52,53].

When Ray et al. [54] studied blood proteins, they found 18 that characterized 90% of people with mild cognitive impairment (MCI), who would 2–6 years later be diagnosed with AD. Mapstone et al. [55] studied blood lipids and found 10, including eight phosphatidylcholine lipids, that correlated with 90% accuracy with the age of onset of MCI and AD diagnosis 2–3 years later.

When Leszek et al. [56] analyzed the serum of AD patients for advanced glycation end-products, they found a novel antigen epitope, which was significantly reduced in AD patients compared to control subjects, whereas the antigen-antibody complexes were elevated in AD patients. This novel epitope was detected with antibodies made in mice against the antigen synthesized in vitro in anhydrous conditions [57]. This antigen epitope may represent a totally different pool of protein adducts, different from known fluorescent advanced glycation end-products. Its structure and function in human physiology and pathology, if any, remains to be elucidated. Further, whether this antigen epitope may serve as a novel AD biomarker remains to be studied.

DIAN (Dominantly Inherited Alzheimer Network) is an international registry of families carrying inherited dominant mutations in the APP, PS1 or PS2 genes, which cause the early-onset familial forms of AD, of 0.5 million people or 1% of AD [58,59,60,61]. DIAN provides an unprecedented, unique opportunity to uncover molecular details and cellular mechanisms at work decades before AD begins.

When Bateman et al. [62] studied cognitively unimpaired people carrying the PS1 mutation E280A, they found many changes in AD biomarkers 10–25 years early to the expected age of onset of AD (age 49 with E280A), such as less Aβ peptides in the cerebrospinal fluid, less glucose uptake and more amyloid in the brain by PET imaging, and more brain atrophy by MRI. These are remarkable findings considering that the people with the E280A mutation were cognitively unimpaired at the study time. Of note, they also found “impaired episodic memory” 10 years ahead of AD. Compared to other AD biomarkers, measuring ‘episodic memory’ is safe, non-invasive, cost-effective, and takes no time.

Recently, Mattsson-Carlgren et al. [63] monitored for six years (at base line) cognitively unimpaired and impaired study participants and found p-tau217 (tau protein phosphorylated at threonine 217) plasma level increased faster in cognitively impaired over the cognitively unimpaired participants. Moreover, p-tau217 increased even faster in cognitively impaired participants who developed AD.

Palmqkvist et al. [64] reported that plasma p-tau217 in AD patients was seven times higher over the control subjects and could distinguish AD from other neurodegenerative brain diseases such as frontotemporal dementia, vascular dementia, and Parkinson’s disease. Plasma p-tau217 correlated with tau PET imaging in patients with amyloid but did not correlate in patients without amyloid. Most impressively, when they studied people carrying the PS1 E280A mutation, they found p-tau217 increased linearly on a logarithmic scale from 1 pg/mL in people at age 25 to 30 pg/mL in people at age 60. In contrast, in people from the same kindred without the mutation, p-tau217 stayed the same at 1 pg/mL. Thus, in the mutation carriers, p-tau217 is forecasting AD 20 years before its expected onset at age 49. It is peculiar, though, that p-tau217 was not increased in people without the mutation, even at age 60. Because of the 10% incidence of AD at age 65, one would expect some of the control subjects of one hundred had shown elevated p-tau217 as a sign of developing AD.

## 5. AD Drug Trials

A popular argument to ‘explain’ the unsuccessful clinical trials in mild to moderate AD patients has been ‘too little too late’. While the drugs reduced Aβ peptide production and brain amyloid, they did slow cognitive decline because, as the argument goes, at the time of intervention, AD had already progressed beyond the point of therapy [65].

Trials with β-secretase inhibitors in cognitively unimpaired people aged 65–85 at high risk for AD due to AP-OE4 or elevated brain amyloid PET scan were all stopped early after 12 months because of the emergence of adverse events and serious health problems, including impaired cognition [66,67,68,69,70]. Similarly, a recent 5-year trial with anti-Aβ antibodies gantenerumab or solanezumab in cognitively unimpaired people carrying the PS1 mutation E280A was terminated due to lack of efficacy in preventing cognitive decline. Solanezumab even enhanced cognitive decline [71].

The US Food and Drug Administration (FDA) on 6 January 2023 approved lecanemab, a monoclonal antibody for the treatment of early Alzheimer’s disease [72]. Lecanemab reduced markers of amyloid in AD and resulted in moderately less decline on measures of cognition and function than placebo at 18 months of treatment slowed cognitive decline by 27% compared with placebo but was associated with adverse events. As reported by van Dyck CH et al. [73], lecanemab slowed cognitive decline by 0.45 points as measured by CDR-SB, an 18-point scale. At the study entry, baseline CDR-SB was 3.2. During the 18-month trial, CDR-SB increased 1.66 points in the control group and 1.21 points in the Lecanemab group, a difference of 0.45 points, or 27% (0.45/1.66). This 27% as the clinical benefit of lecanemab treatment can be misleading and a wrong comparison. What matters at the end of the trial is the difference in the total CDR-SB (which includes the 3.2 baseline), that is, 4.86 in the control group and 4.41 in the lecanemab group, or 9.3% (0.45/4.86). 9.3% is unlikely to provide clinically meaningful benefit in people living with mild to moderate Alzheimer’s disease.

It is fair to say, the absence of disease-modifying drug treatments for AD today is due to the amyloid cascade hypothesis, a misguided hypothesis of AD etiology, its origin, and disease mechanisms which has dominated drug discovery and clinical development in AD for 30 years. However, we must learn what the clinical trial failures can teach us about AD. The drugs engaged their intended targets, as measured by reduced Aβ peptide production and brain amyloid formation. Discovering the ‘off-targets’ engaged by the drugs, and the effects thereof, could help uncover causes for the trial failures, which in turn, could lead to better AD drugs and much-needed success in preventive trial studies in AD.

## 6. The Presenilin Hypothesis

Presenilin PS1 is the proteolytic enzyme component of γ-secretase, which cuts APP to produce Aβ peptides which form amyloid in the brain. Mutations in PS1 are the most common cause of early-onset AD. According to the amyloid cascade hypothesis of AD, Aβ peptides and brain amyloid formation cause neurodegeneration and dementia. As discussed above, many observations and facts are against the amyloid hypothesis.

The presenilin hypothesis of AD by Shen and Kelleher [74] suggests that loss of essential functions of PS1 is associated with neurodegeneration and dementia. This is a strikingly novel idea, because it calls for AD treatment by increasing γ-secretase activity, rather than decreasing it, as has been done in the past.

The presenilin hypothesis is derived from a few key observations. Inactivation of one copy of the PS1 gene in the adult mouse brain causes progressive neurodegeneration and memory loss, whereas mice overproducing Aβ peptides in the brain have no neurodegeneration. Pathogenic PS1 mutations which increase Aβ42 production also decrease Aβ40 production and impair other PS1-dependent events. Inhibitors of γ-secretase can enhance the production of Aβ42 while reducing other γ-secretase activities, thus mimicking the effects of pathogenic PS1 mutations.

**Figure 1:**
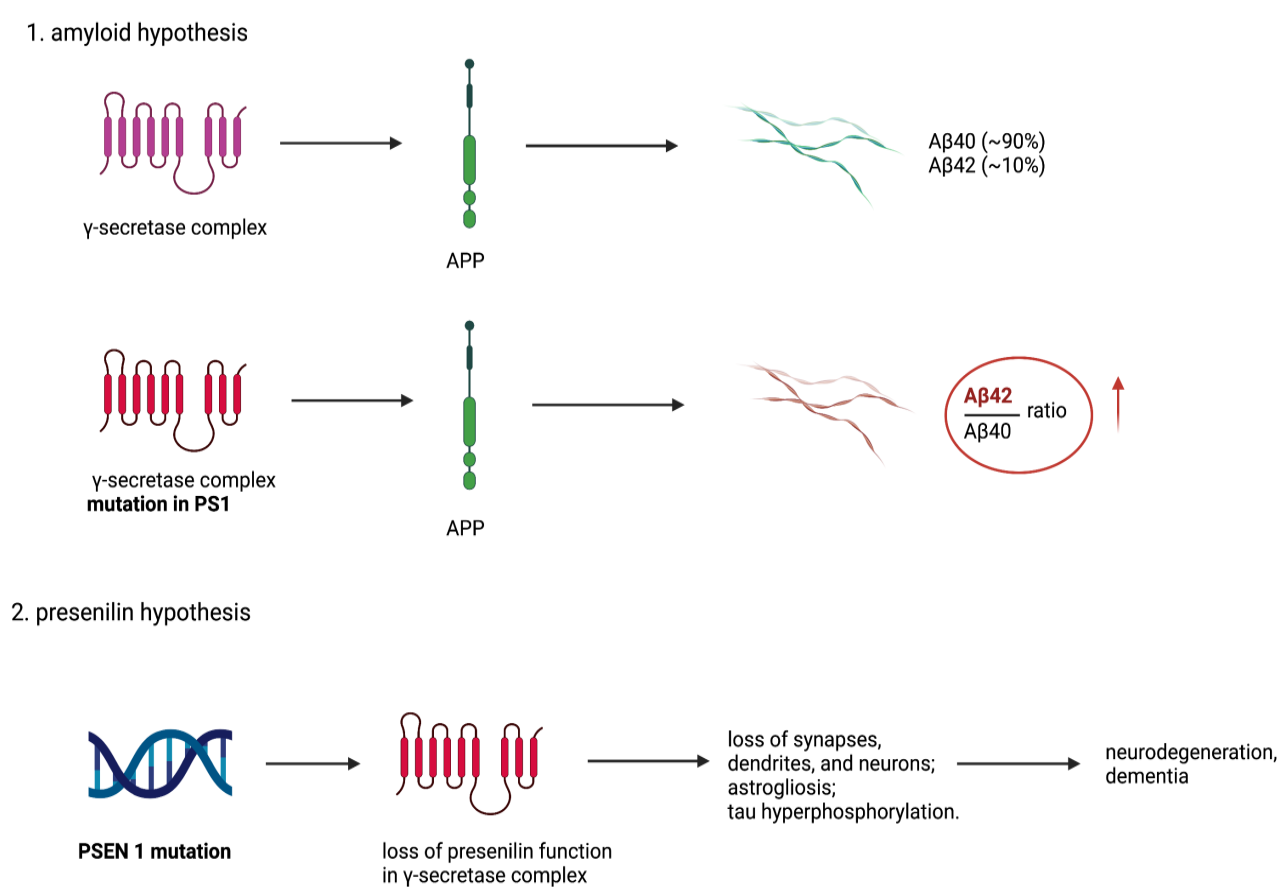
The comparison between amyloid and presenilin hypotheses. The amyloid hypothesis proposed that mutation in PSEN1 results in presenilin dysfunction, thus change in γ-secretase activity resulting in relative increase of the Aβ42/Aβ40 ratio, which is significantly increased in AD pathologies. The presenilin hypothesis does not exclude the amyloid hypothesis but complements it. It presents theory that PSEN1 mutation and following loss of presenilin normal activity in brain triggers neurodegeneration and dementia also without coexisting amyloid formation. Created with BioRender.com.

When Sun et al. [34] studied 138 pathogenic PS1 mutations on the in vitro production of the Aβ42 and Aβ40 peptides by γ-secretase, they could not find any correlation between the amount of Aβ peptides produced or the Aβ42/40 ratio and the age of onset of AD (Figure 1). Remarkably, one third of the PS1 mutations produced no Aβ peptides (and yet they dominate in causing AD at different ages of onset). This observation agrees with the study (referred to above) in which inactivation of one PS1 gene in the adult mouse brain caused neurodegeneration and progressive memory loss. As more evidence for the presenilin hypothesis, a loss-of-function pathogenic mechanism of PS1 in AD is generated; it already seems clear it does not involve Aβ peptides and brain amyloid. Cataloguing PS1 interacting proteins and target substrates should help find many events regulated by PS1. One of the proteins is glutamate transporter EAAT2 [75], and there are 149 substrates for PS1 [76].

## 7. Synaptic Glutamate Signaling

The human brain has 86 billion neurons, 85 billion glia cells (astrocytes, oligodendrocytes, and microglia), and 1000 trillion synapses [77,78]. Synapses are covered by astrocytes, which play an essential role in synapse formation, synaptic activity, and neural circuit development [79,80,81,82,83,84,85].

A total of 85% of neurotransmission is excitatory. Glutamate mediates 95% of excitatory signaling; the remaining 5% is acetylcholine, dopamine, glycine, histamine and serotonin signaling. Fifteen percent of neurotransmission is inhibitory, mediated by GABA (γ-amino butyric acid) derived from glutamate. Excitatory synapses target dendrite spines, and inhibitory synapses target the neuron cell body. 

In the neural circuit, neurons are subject to both excitatory and inhibitory input, synchronized and connected in their activity [85,86]. Brain activity, as studied by EEG or MEG, appears differently synchronized and connected in AD patients compared to cognitively unimpaired people, and studies have revealed hippocampal hyperactivation in mild cognitive impairment and in asymptomatic early-onset AD [87,88,89,90,91,92]. 

Synaptic glutamate signaling begins when glutamate, released from the nerve ending, enters the synaptic cleft and binds to postsynaptic glutamate membrane receptors. This allows Ca^2+^ inflow, which initiates calcium-regulated signaling events in the postsynaptic neuron [93,94,95,96,97]. However, as soon as the glutamate signaling starts it is stopped in 1 ms by astrocytes, which take up and clear glutamate from the synapses. This makes the glutamate signaling essentially an on-or-off event, necessary for a high-speed neurotransmission with precision [98,99,100]. Fast glutamate clearance also prevents ‘excitotoxicity’ caused by extended glutamate signaling and excessive Ca^2+^ inflow and calcium signaling, which can impair synaptic structure and function and cause synapse loss and, in the end, neuron cell death.

## 8. Astrocyte Glutamate Transporter EAAT2

Humans have five glutamate transporters, also called excitatory amino acid transporter (EAAT), which differ in tissue and cell distribution, sub-cellular location, and glutamate uptake kinetics [101,102,103]. Astrocytes express most of the glutamate transporter EAAT2, representing 1% of brain protein and 95% of synaptic glutamate uptake [104]. By electron microscope studies, 90% of EAAT2 is found in the perisynaptic astrocyte membrane processes around synapses [105]. As described in more detail elsewhere [106], in transgenic mouse models of AD, increasing EAAT2 expression slows disease progression, and decreasing EAAT2 expression enhances disease progression. There is less EAAT2 in the human AD brain. Here are a few examples:

When APPswe/ind mice (which express 40% less EAAT2 in the brain) were crossed with transgenic EAAT2 mice expressing 2-fold more EAAT2, EAAT2 expression was normalized in the crossed mice, which also showed improved “cognitive functions, restored synaptic integrity, and reduced amyloid plaques” [107]. Moreover, in APPswe, Ind mice, a drug-induced increase of EAAT2 protein (by increased mRNA translation) improved “cognitive functions, restored synaptic integrity, and reduced amyloid plaques.” These effects were not seen if EAAT2 was inhibited with dihydrokainate. Even after stopping the drug treatment, the effects were observed for one month, which prompted the authors to write: “EAAT2 is a potential disease modifier with therapeutic potential for AD” [108].

When APPswe/PS1dE9 mice were crossed with transgenic mice carrying only one EAAT2 gene, the crossed mice with one EAAT2 gene, showed increased spatial memory problems at 6 months and behavioral disorders at 9 months. These results suggest that impaired synaptic glutamate uptake (due to reduced EAAT2 expression) enhances the progression of AD caused by APP and PS1 mutations [109].

In a study of midfrontal cortex of postmortem brains of AD patients, levels of EAAT2 (measured by [3H]aspartate binding), synaptophysin, and brain spectrin degradation products were compared to brains of control subjects. In comparison to control brains, AD brains had 30% less [3H]aspartate binding, 48% less of synapto-physin, and increased levels of brain spectrin degradation products. These results suggest that decreased EAAT2 activity in AD is associated with increased synaptic damage and neurodegeneration [110]. 

As shown by Jacob and colleagues [111], in a study of EAAT1, EAAT2 and glutamate receptors in AD brains, EAAT1 and EAAT2 gene and protein expression were already reduced in the early stages of AD progression, in hippocampus in gyrus frontalis medialis. The loss of EAAT1 and EAAT2 proteins was particularly obvious in the vicinity of amyloid plaques. This study supports the causal role of impaired synaptic glutamate uptake and glutamate ‘excitotoxicity’ in the pathogenesis of AD dementia. 

These examples indicate EAAT2 as a novel target for disease-modifying therapies in AD. Discovery and clinical development of drugs increasing EAAT2 expression or activity could offer new treatments to prevent or slow AD progression [112,113,114]. Interestingly, Falcucci et al. [115] have discovered a small-molecule drug (GT949), which in astrocyte-neuron cell culture protects neurons against glutamate ‘excitotoxicity’.

## 9. In Perspective

The amyloid cascade hypothesis has almost singularly guided AD research, drug development, and clinical trials by targeting Aβ peptides and brain amyloid. Inherited dominant mutations in APP, PS1 or PS2 cause early-onset AD, and yet, after 30 years of research, we know little about the molecular and cellular mechanisms of their action in the development of AD. Even though clinical trial studies with Aβ amyloid-lowering drugs have been nothing but failures in slowing cognitive decline, and often times only harmed the study participants volunteering for the trials, the hypothesis is not dead yet, as exemplified by the FDA approval, on 7 June 2021, of aducanumab (an anti-Aβ antibody) for the treatment of AD [116]. 

Naturally, of course, APP is more than the precursor protein for Aβ peptides, and Aβ peptides do much more than form amyloid, as discussed in more detail elsewhere [106]. For example, APP is a G protein-coupled receptor (GPCR) important in learning and memory [117]. In embryonic brain development, APP is involved in neural circuit formation, by the elimination of synapses, dendrites, axons, and neurons [118]. APP plays a role in cancer progression and metastasis formation [119], and APP inhibits proteinases [120]. APP and Aβ peptides are involved in cerebral hemostasis, capillary blood flow, thrombotic and fibrinolytic events, and hemorrhagic and ischemic strokes [121,122]. Aβ peptides are potent wide-spectrum antimicrobial peptides, an ancient arm of the innate immune system against infections by bacteria, fungi, and viruses [123,124]. 

Development of AD takes time, 65, 75, 85 or more years before the signs and symptoms appear, as if it could be progressing at different paces. Considering that development of AD happens in many steps, one of which is the rate-limiting step, removing that step would introduce another rate-limiting step, which (the way rate-limiting steps work) would increase the pace of AD development. After removing that step, there would be another step doing the same. Is there a rate-limiting step in the development of Alzheimer’s dementia? [106].

A case in point here is the E280A mutation of PS1, which causes AD at age 49. Recent studies by Vélez et al. [125] have discovered three gene variants in people carrying the E280A mutation, which acted recessively to delay the age of onset of AD by 6–11 years, and one gene variant which accelerated the age of onset by 8 years. It seems, these recessive gene variants can interfere with the pathogenic mechanism of action of the PS1 E280A mutant (which is not known), by decreasing or increasing the pace of AD development. In another study, Vélez et al. [126] found that two copies of the APOE2 gene could delay AD onset by 11 years in the E280A mutant carriers. 

Most strikingly, however, a recent case report [127] describes an elderly woman in Christchurch, New Zealand, with the E280A mutation and an unusually high amount of brain amyloid yet experiencing no cognitive impairment until her mid-seventies. She also had two copies of the APOE3Christchurch mutation R136S, which apparently had been protecting her for 30 years against the PS1 mutant E280A.

The significance of these observations is obvious. Studies on the mechanism of action of these recessive gene variants in slowing the pace of AD development may help in finding drugs that can do the same and delay the onset of AD [125]. Such drugs, in combination with the awareness of lifestyle risk factors [49,50], will provide better prevention and treatment of AD.

Today, AD is an incurable disease. Fifty million people are living with AD, and hundreds of millions of family members, friends, physicians, and professional caregivers are living with AD patients. What we need now is a dementia-friendly society, accessible to everyone.

## Data Availability

Not applicable.

## References

[1] Franceschi C., Garagnani P., Morsiani C., Conte M., Santoro A., Grignolio A., Monti D., Capri M., Salvioli S. (2018). The continuum of aging and age-related diseases: Common mechanisms but different rates. Front. Med..

[2] Alzheimer A. (1907). Uber eine eigenartige Erkrankung der Hirnrinde. Zentralbl. Nervenh. Psych..

[3] Bomilcar I., Bertrand E., Morris R.G., Mograbi D.C. (2021). The seven selves of dementia. Front. Psychiatry.

[4] Whitehouse P., George D. (2008). The Myth of Alzheimer’s.

[5] Qiu C., Kivipelto M., von Strauss E. (2009). Epidemiology of Alzheimer’s disease: Occurrence, determinants, and strategies toward intervention. Dialogues Clin. Neurosci..

[6] Lane C.A., Hardy J., Schott J.M. (2018). Alzheimer’s disease. Eur. J. Neurol..

[7] Roses A.D. (1996). Apolipoprotein E alleles as risk factors in Alzheimer’s disease. Annu. Rev. Med..

[8] Tanzi R.E. (2013). A brief history of Alzheimer’s disease gene discovery. J. Alzheimers Dis..

[9] Karch C.M., Goate A.M. (2015). Alzheimer’s disease risk genes and mechanisms of disease pathogenesis. Biol. Psychiatry.

[10] Van Cauwenberghe C., Van Broeckhoven C., Sleegers K. (2016). The genetic landscape of Alzheimer disease: Clinical implications and perspectives. Genet. Med..

[11] Martens Y.A., Zhao N., Liu C.-C., Kanekiyo T., Yang A.J., Goate A.M., Holtzman D.M., Bu G. (2022). ApoE Cascade Hypothesis in the pathogenesis of Alzheimer’s disease and related dementias. Neuron.

[12] International Alzheimer’s Disease Numbers of People with Dementia Worldwide. https://www.alzint.org/resource/world-alzheimer-report-2015/.

[13] Alzheimer’s Association (2021). 2021 Alzheimer’s disease facts and figures. Alzheimers Dement..

[14] Brookmeyer R., Abdalla N., Kawas C.H., Corrada M.M. (2018). Forecasting the prevalence of preclinical and clinical Alzheimer’s disease in the United States. Alzheimers Dement..

[15] NIH Bypass Budget Proposal for Fiscal Year 2021. https://www.nia.nih.gov/sites/default/files/2019-07/FY21-bypass-budget-report-508.pdf.

[16] Yuan X.-Z., Sun S., Tan C.-C., Yu J.-T., Tan L. (2017). The Role of ADAM10 in Alzheimer’s Disease. J. Alzheimers Dis..

[17] Schreiner B., Hedskog L., Wiehager B., Ankarcrona M. (2015). Amyloid-β peptides are generated in mitochondria-associated endoplasmic reticulum membranes. J. Alzheimers Dis..

[18] Ben Halima S., Mishra S., Raja K.M.P., Willem M., Baici A., Simons K., Brüstle O., Koch P., Haass C., Caflisch A. (2016). Specific inhibition of β-secretase processing of the Alzheimer disease amyloid precursor protein. Cell Rep..

[19] Xia W. (2019). γ-Secretase and its modulators: Twenty years and beyond. Neurosci. Lett..

[20] Liu L., Ding L., Rovere M., Wolfe M.S., Selkoe D.J. (2019). A cellular complex of BACE1 and γ-secretase sequentially generates Aβ from its full-length precursor. J. Cell Biol..

[21] Müller U.C., Deller T., Korte M. (2017). Not just amyloid: Physiological functions of the amyloid precursor protein family. Nat. Rev. Neurosci..

[22] Järemo P., Jejcic A., Jelic V., Shahnaz T., Oweling M., Winblad B., Behbahani H. (2019). erythrocyte amyloid beta peptide isoform distributions in alzheimer and mild cognitive impairment. Curr. Alzheimer Res..

[23] Bush A.I., Martins R.N., Rumble B., Moir R., Fuller S., Milward E., Currie J., Ames D., Weidemann A., Fischer P. (1990). The amyloid precursor protein of Alzheimer’s disease is released by human platelets. J. Biol. Chem..

[24] Mönning U., König G., Prior R., Mechler H., Schreiter-Gasser U., Masters C.L., Beyreuther K. (1990). Synthesis and secretion of Alzheimer amyloid beta A4 precursor protein by stimulated human peripheral blood leucocytes. FEBS Lett..

[25] Hardy J. (2017). The discovery of Alzheimer-causing mutations in the APP gene and the formulation of the “amyloid cascade hypothesis”. FEBS J..

[26] Kang J., Lemaire H.G., Unterbeck A., Salbaum J.M., Masters C.L., Grzeschik K.H., Multhaup G., Beyreuther K., Müller-Hill B. (1987). The precursor of Alzheimer’s disease amyloid A4 protein resembles a cell-surface receptor. Nature.

[27] Hardy J., Allsop D. (1991). Amyloid deposition as the central event in the aetiology of Alzheimer’s disease. Trends Pharmacol. Sci..

[28] Hardy J.A., Higgins G.A. (1992). Alzheimer’s disease: The amyloid cascade hypothesis. Science.

[29] Hardy J., Selkoe D.J. (2002). The amyloid hypothesis of Alzheimer’s disease: Progress and problems on the road to therapeutics. Science.

[30] Selkoe D.J., Hardy J. (2016). The amyloid hypothesis of Alzheimer’s disease at 25 years. EMBO Mol. Med..

[31] Karran E., De Strooper B. (2016). The amyloid cascade hypothesis: Are we poised for success or failure?. J. Neurochem..

[32] Bertram L., Lill C.M., Tanzi R.E. (2010). The genetics of Alzheimer disease: Back to the future. Neuron.

[33] Rosenberg R.N., Lambracht-Washington D., Yu G., Xia W. (2016). Genomics of Alzheimer Disease: A Review. JAMA Neurol..

[34] Sun L., Zhou R., Yang G., Shi Y. (2017). Analysis of 138 pathogenic mutations in presenilin-1 on the in vitro production of Aβ42 and Aβ40 peptides by γ-secretase. Proc. Natl. Acad. Sci. USA.

[35] Vemuri P., Weigand S.D., Przybelski S.A., Knopman D.S., Smith G.E., Trojanowski J.Q., Shaw L.M., Decarli C.S., Carmichael O., Bernstein M.A. (2011). Cognitive reserve and Alzheimer’s disease biomarkers are independent determinants of cognition. Brain.

[36] Rosenberg R.N. (2015). Defining amyloid pathology in persons with and without dementia syndromes: Making the right diagnosis. JAMA.

[37] Mountz J.M., Laymon C.M., Cohen A.D., Zhang Z., Price J.C., Boudhar S., McDade E., Aizenstein H.J., Klunk W.E., Mathis C.A. (2015). Comparison of qualitative and quantitative imaging characteristics of [11C]PiB and [18F]flutemetamol in normal control and Alzheimer’s subjects. NeuroImage. Clin..

[38] Price J.L., Morris J.C. (1999). Tangles and plaques in nondemented aging and “preclinical” Alzheimer’s disease. Ann. Neurol..

[39] Knopman D.S., Parisi J.E., Salviati A., Floriach-Robert M., Boeve B.F., Ivnik R.J., Smith G.E., Dickson D.W., Johnson K.A., Petersen L.E. (2003). Neuropathology of cognitively normal elderly. J. Neuropathol. Exp. Neurol..

[40] Snowdon D.A. (1997). Aging and Alzheimer’s disease: Lessons from the Nun Study. Gerontologist.

[41] Sevigny J., Chiao P., Bussière T., Weinreb P.H., Williams L., Maier M., Dunstan R., Salloway S., Chen T., Ling Y. (2017). Addendum: The antibody aducanumab reduces Aβ plaques in Alzheimer’s disease. Nature.

[42] Cummings J., Aisen P., Lemere C., Atri A., Sabbagh M., Salloway S. (2021). Aducanumab produced a clinically meaningful benefit in association with amyloid lowering. Alzheimers Res. Ther..

[43] Chia C.W., Egan J.M., Ferrucci L. (2018). Age-Related Changes in Glucose Metabolism, Hyperglycemia, and Cardiovascular Risk. Circ. Res..

[44] Parsons C.G., Danysz W., Dekundy A., Pulte I. (2013). Memantine and cholinesterase inhibitors: Complementary mechanisms in the treatment of Alzheimer’s disease. Neurotox. Res..

[45] Khoury R., Rajamanickam J., Grossberg G.T. (2018). An update on the safety of current therapies for Alzheimer’s disease: Focus on rivastigmine. Ther. Adv. drug Saf..

[46] Gąsiorowski K., Brokos J.B., Sochocka M., Ochnik M., Chojdak-Łukasiewicz J., Zajączkowska K., Fułek M., Leszek J. (2022). Current and Near-Future Treatment of Alzheimer’s Disease. Curr. Neuropharmacol..

[47] Blennow K., Zetterberg H. (2018). The Past and the Future of Alzheimer’s Disease Fluid Biomarkers. J. Alzheimers Dis..

[48] Lloret A., Esteve D., Lloret M.-A., Cervera-Ferri A., Lopez B., Nepomuceno M., Monllor P. (2019). When Does Alzheimer’s Disease Really Start? The Role of Biomarkers. Int. J. Mol. Sci..

[49] Hrubešová K., Fousková M., Habartová L., Fišar Z., Jirák R., Raboch J., Setnička V. (2019). Search for biomarkers of Alzheimer’s disease: Recent insights, current challenges and future prospects. Clin. Biochem..

[50] Yu J.-T., Xu W., Tan C.-C., Andrieu S., Suckling J., Evangelou E., Pan A., Zhang C., Jia J., Feng L. (2020). Evidence-based prevention of Alzheimer’s disease: Systematic review and meta-analysis of 243 observational prospective studies and 153 randomised controlled trials. J. Neurol. Neurosurg. Psychiatry.

[51] Kivipelto M., Mangialasche F., Snyder H.M., Allegri R., Andrieu S., Arai H., Baker L., Belleville S., Brodaty H., Brucki S.M. (2020). World-Wide FINGERS Network: A global approach to risk reduction and prevention of dementia. Alzheimers Dement..

[52] Dubois B., Villain N., Frisoni G.B., Rabinovici G.D., Sabbagh M., Cappa S., Bejanin A., Bombois S., Epelbaum S., Teichmann M. (2021). Clinical diagnosis of Alzheimer’s disease: Recommendations of the International Working Group. Lancet. Neurol..

[53] Jack C.R.J., Bennett D.A., Blennow K., Carrillo M.C., Dunn B., Haeberlein S.B., Holtzman D.M., Jagust W., Jessen F., Karlawish J. (2018). NIA-AA research framework: Toward a biological definition of Alzheimer’s disease. Alzheimers Dement..

[54] Ray S., Britschgi M., Herbert C., Takeda-Uchimura Y., Boxer A., Blennow K., Friedman L.F., Galasko D.R., Jutel M., Karydas A. (2007). Classification and prediction of clinical Alzheimer’s diagnosis based on plasma signaling proteins. Nat. Med..

[55] Mapstone M., Cheema A.K., Fiandaca M.S., Zhong X., Mhyre T.R., MacArthur L.H., Hall W.J., Fisher S.G., Peterson D.R., Haley J.M. (2014). Plasma phospholipids identify antecedent memory impairment in older adults. Nat. Med..

[56] Leszek J., Malyszczak K., Bartys A., Staniszewska M., Gamian A. (2006). Analysis of serum of patients with Alzheimer’s disease for the level of advanced glycation end products. Am. J. Alzheimers Dis. Other Demen..

[57] Staniszewska M., Bronowicka-Szydełko A., Gostomska-Pampuch K., Szkudlarek J., Bartyś A., Bieg T., Gamian E., Kochman A., Picur B., Pietkiewicz J. (2021). The melibiose-derived glycation product mimics a unique epitope present in human and animal tissues. Sci. Rep..

[58] Morris J.C., Aisen P.S., Bateman R.J., Benzinger T.L.S., Cairns N.J., Fagan A.M., Ghetti B., Goate A.M., Holtzman D.M., Klunk W.E. (2012). Developing an international network for Alzheimer research: The Dominantly Inherited Alzheimer Network. Clin. Investig..

[59] Ryman D.C., Acosta-Baena N., Aisen P.S., Bird T., Danek A., Fox N.C., Goate A., Frommelt P., Ghetti B., Langbaum J.B.S. (2014). Symptom onset in autosomal dominant Alzheimer disease: A systematic review and meta-analysis. Neurology.

[60] Bateman R.J., Benzinger T.L., Berry S., Clifford D.B., Duggan C., Fagan A.M., Fanning K., Farlow M.R., Hassenstab J., McDade E.M. (2017). The DIAN-TU Next Generation Alzheimer’s prevention trial: Adaptive design and disease progression model. Alzheimers Dement..

[61] Lopez Lopez C., Tariot P.N., Caputo A., Langbaum J.B., Liu F., Riviere M.-E., Langlois C., Rouzade-Dominguez M.-L., Zalesak M., Hendrix S. (2019). The Alzheimer’s Prevention Initiative Generation Program: Study design of two randomized controlled trials for individuals at risk for clinical onset of Alzheimer’s disease. Alzheimer’s Dement..

[62] Bateman R.J., Xiong C., Benzinger T.L.S., Fagan A.M., Goate A., Fox N.C., Marcus D.S., Cairns N.J., Xie X., Blazey T.M. (2012). Clinical and biomarker changes in dominantly inherited Alzheimer’s disease. N. Engl. J. Med..

[63] Mattsson-Carlgren N., Janelidze S., Palmqvist S., Cullen N., Svenningsson A.L., Strandberg O., Mengel D., Walsh D.M., Stomrud E., Dage J.L. (2020). Longitudinal plasma p-tau217 is increased in early stages of Alzheimer’s disease. Brain.

[64] Palmqvist S., Janelidze S., Quiroz Y.T., Zetterberg H., Lopera F., Stomrud E., Su Y., Chen Y., Serrano G.E., Leuzy A. (2020). Discriminative Accuracy of Plasma Phospho-tau217 for Alzheimer Disease vs Other Neurodegenerative Disorders. JAMA.

[65] McDade E., Llibre-Guerra J.J., Holtzman D.M., Morris J.C., Bateman R.J. (2021). The informed road map to prevention of Alzheimer Disease: A call to arms. Mol. Neurodegener..

[66] Kurkinen M. (2017). The amyloid hypothesis is too good to be true. Alzheimer’s Dement. Cogn. Neurol..

[67] Mullard A. (2018). BACE failures lower AD expectations, again. Nat. Rev. Drug Discov..

[68] Henley D., Raghavan N., Sperling R., Aisen P., Raman R., Romano G. (2019). Preliminary Results of a Trial of Atabecestat in Preclinical Alzheimer’s Disease. N. Engl. J. Med..

[69] Egan M.F., Mukai Y., Voss T., Kost J., Stone J., Furtek C., Mahoney E., Cummings J.L., Tariot P.N., Aisen P.S. (2019). Further analyses of the safety of verubecestat in the phase 3 EPOCH trial of mild-to-moderate Alzheimer’s disease. Alzheimers Res. Ther..

[70] Yiannopoulou K.G., Anastasiou A.I., Zachariou V., Pelidou S.-H. (2019). Reasons for Failed trials of disease-modifying treatments for Alzheimer disease and their contribution in recent research. Biomedicines.

[71] Imbimbo B.P., Lucca U., Watling M. (2021). Can Anti-β-amyloid Monoclonal Antibodies Work in Autosomal Dominant Alzheimer Disease?. Neurol. Genet..

[72] FDA Grants Accelerated Approval for Alzheimer’s Disease Treatment. https://www.fda.gov/news-events/press-announcements/fda-grants-accelerated-approval-alzheimers-disease-treatment.

[73] van Dyck C.H., Swanson C.J., Aisen P., Bateman R.J., Chen C., Gee M., Kanekiyo M., Li D., Reyderman L., Cohen S. (2023). Lecanemab in Early Alzheimer’s Disease. N. Engl. J. Med..

[74] Shen J., Kelleher R.J. (2007). 3rd The presenilin hypothesis of Alzheimer’s disease: Evidence for a loss-of-function pathogenic mechanism. Proc. Natl. Acad. Sci. USA.

[75] Zoltowska K.M., Maesako M., Meier J., Berezovska O. (2018). Novel interaction between Alzheimer’s disease-related protein presenilin 1 and glutamate transporter 1. Sci. Rep..

[76] Güner G., Lichtenthaler S.F. (2020). The substrate repertoire of γ-secretase/presenilin. Semin. Cell Dev. Biol..

[77] Azevedo F.A.C., Carvalho L.R.B., Grinberg L.T., Farfel J.M., Ferretti R.E.L., Leite R.E.P., Jacob Filho W., Lent R., Herculano-Houzel S. (2009). Equal numbers of neuronal and nonneuronal cells make the human brain an isometrically scaled-up primate brain. J. Comp. Neurol..

[78] Alonso-Nanclares L., Gonzalez-Soriano J., Rodriguez J.R., DeFelipe J. (2008). Gender differences in human cortical synaptic density. Proc. Natl. Acad. Sci. USA.

[79] Clarke L.E., Barres B.A. (2013). Emerging roles of astrocytes in neural circuit development. Nat. Rev. Neurosci..

[80] Xu W., Südhof T.C. (2013). A neural circuit for memory specificity and generalization. Science.

[81] Heller J.P., Rusakov D.A. (2015). Morphological plasticity of astroglia: Understanding synaptic microenvironment. Glia.

[82] Allen N.J., Eroglu C. (2017). Cell Biology of Astrocyte-Synapse Interactions. Neuron.

[83] Papouin T., Dunphy J., Tolman M., Foley J.C., Haydon P.G. (2017). Astrocytic control of synaptic function. Philos. Trans. R. Soc. London. Ser. B Biol. Sci..

[84] Verkhratsky A., Nedergaard M. (2018). Physiology of astroglia. Physiol. Rev..

[85] Südhof T.C. (2018). Towards an understanding of synapse formation. Neuron.

[86] Froemke R.C. (2015). Plasticity of cortical excitatory-inhibitory balance. Annu. Rev. Neurosci..

[87] Sohal V.S., Rubenstein J.L.R. (2019). Excitation-inhibition balance as a framework for investigating mechanisms in neuropsychiatric disorders. Mol. Psychiatry.

[88] Babiloni C., Blinowska K., Bonanni L., Cichocki A., De Haan W., Del Percio C., Dubois B., Escudero J., Fernández A., Frisoni G. (2020). What electrophysiology tells us about Alzheimer’s disease: A window into the synchronization and connectivity of brain neurons. Neurobiol. Aging.

[89] Wisch J.K., Roe C.M., Babulal G.M., Schindler S.E., Fagan A.M., Benzinger T.L., Morris J.C., Ances B.M. (2020). Resting State Functional Connectivity Signature Differentiates Cognitively Normal from Individuals Who Convert to Symptomatic Alzheimer’s Disease. J. Alzheimers Dis..

[90] Dickerson B.C., Salat D.H., Greve D.N., Chua E.F., Rand-Giovannetti E., Rentz D.M., Bertram L., Mullin K., Tanzi R.E., Blacker D. (2005). Increased hippocampal activation in mild cognitive impairment compared to normal aging and AD. Neurology.

[91] Quiroz Y.T., Budson A.E., Celone K., Ruiz A., Newmark R., Castrillón G., Lopera F., Stern C.E. (2010). Hippocampal hyperactivation in presymptomatic familial Alzheimer’s disease. Ann. Neurol..

[92] Busche M.A., Konnerth A. (2015). Neuronal hyperactivity--A key defect in Alzheimer’s disease?. Bioessays.

[93] Zott B., Busche M.A., Sperling R.A., Konnerth A. (2018). What Happens with the Circuit in Alzheimer’s Disease in Mice and Humans?. Annu. Rev. Neurosci..

[94] Bronner F. (2001). Extracellular and intracellular regulation of calcium homeostasis. ScientificWorldJournal..

[95] Clapham D.E. (2007). Calcium signaling. Cell.

[96] Südhof T.C. (2013). Neurotransmitter release: The last millisecond in the life of a synaptic vesicle. Neuron.

[97] Zheng K., Jensen T.P., Savtchenko L.P., Levitt J.A., Suhling K., Rusakov D.A. (2017). Nanoscale diffusion in the synaptic cleft and beyond measured with time-resolved fluorescence anisotropy imaging. Sci. Rep..

[98] Clements J.D., Lester R.A., Tong G., Jahr C.E., Westbrook G.L. (1992). The time course of glutamate in the synaptic cleft. Science.

[99] Herman M.A., Jahr C.E. (2007). Extracellular glutamate concentration in hippocampal slice. J. Neurosci..

[100] Scimemi A., Beato M. (2009). Determining the neurotransmitter concentration profile at active synapses. Mol. Neurobiol..

[101] Vandenberg R.J., Ryan R.M. (2013). Mechanisms of glutamate transport. Physiol. Rev..

[102] Murphy-Royal C., Dupuis J., Groc L., Oliet S.H.R. (2017). Astroglial glutamate transporters in the brain: Regulating neurotransmitter homeostasis and synaptic transmission. J. Neurosci. Res..

[103] Olivares-Bañuelos T.N., Chí-Castañeda D., Ortega A. (2019). Glutamate transporters: Gene expression regulation and signaling properties. Neuropharmacology.

[104] Danbolt N.C. (2001). Glutamate uptake. Prog. Neurobiol..

[105] Roberts R.C., Roche J.K., McCullumsmith R.E. (2014). Localization of excitatory amino acid transporters EAAT1 and EAAT2 in human postmortem cortex: A light and electron microscopic study. Neuroscience.

[106] Kurkinen M., Pavlovic Z.M. (2022). Glutamate and Neuropsychiatric Disorders—Current and Emerging Treatments.

[107] Lin C.-L.G., Kong Q., Cuny G.D., Glicksman M.A. (2012). Glutamate transporter EAAT2: A new target for the treatment of neurodegenerative diseases. Future Med. Chem..

[108] Kong Q., Chang L.-C., Takahashi K., Liu Q., Schulte D.A., Lai L., Ibabao B., Lin Y., Stouffer N., Das Mukhopadhyay C. (2014). Small-molecule activator of glutamate transporter EAAT2 translation provides neuroprotection. J. Clin. Investig..

[109] Mookherjee P., Green P.S., Watson G.S., Marques M.A., Tanaka K., Meeker K.D., Meabon J.S., Li N., Zhu P., Olson V.G. (2011). GLT-1 loss accelerates cognitive deficit onset in an Alzheimer’s disease animal model. J. Alzheimers Dis..

[110] Masliah E., Alford M., DeTeresa R., Mallory M., Hansen L. (1996). Deficient glutamate transport is associated with neurodegeneration in Alzheimer’s disease. Ann. Neurol..

[111] Jacob C.P., Koutsilieri E., Bartl J., Neuen-Jacob E., Arzberger T., Zander N., Ravid R., Roggendorf W., Riederer P., Grünblatt E. (2007). Alterations in expression of glutamatergic transporters and receptors in sporadic Alzheimer’s disease. J. Alzheimers Dis..

[112] Takahashi K., Foster J.B., Lin C.-L.G. (2015). Glutamate transporter EAAT2: Regulation, function, and potential as a therapeutic target for neurological and psychiatric disease. Cell. Mol. Life Sci..

[113] Fontana A.C.K. (2015). Current approaches to enhance glutamate transporter function and expression. J. Neurochem..

[114] Pajarillo E., Rizor A., Lee J., Aschner M., Lee E. (2019). The role of astrocytic glutamate transporters GLT-1 and GLAST in neurological disorders: Potential targets for neurotherapeutics. Neuropharmacology.

[115] Falcucci R.M., Wertz R., Green J.L., Meucci O., Salvino J., Fontana A.C.K. (2019). Novel Positive Allosteric Modulators of Glutamate Transport Have Neuroprotective Properties in an in Vitro Excitotoxic Model. ACS Chem. Neurosci..

[116] FDA Grants Accelerated Approval for Alzheimer’s Drug. https://www.fda.gov/news-events/press-announcements/fda-grants-accelerated-approval-alzheimers-drug?utm_medium=email&utm_source=govdelivery.

[117] Deyts C., Clutter M., Pierce N., Chakrabarty P., Ladd T.B., Goddi A., Rosario A.M., Cruz P., Vetrivel K., Wagner S.L. (2019). APP-Mediated Signaling Prevents Memory Decline in Alzheimer’s Disease Mouse Model. Cell Rep..

[118] Nikolaev A., McLaughlin T., O’Leary D.D.M., Tessier-Lavigne M. (2009). APP binds DR6 to trigger axon pruning and neuron death via distinct caspases. Nature.

[119] Strilic B., Yang L., Albarrán-Juárez J., Wachsmuth L., Han K., Müller U.C., Pasparakis M., Offermanns S. (2016). Tumour-cell-induced endothelial cell necroptosis via death receptor 6 promotes metastasis. Nature.

[120] Xu F., Davis J., Hoos M., Van Nostrand W.E. (2017). Mutation of the Kunitz-type proteinase inhibitor domain in the amyloid β-protein precursor abolishes its anti-thrombotic properties in vivo. Thromb. Res..

[121] Van Nostrand W.E. (2016). The influence of the amyloid ß-protein and its precursor in modulating cerebral hemostasis. Biochim. Biophys. Acta.

[122] Nortley R., Korte N., Izquierdo P., Hirunpattarasilp C., Mishra A., Jaunmuktane Z., Kyrargyri V., Pfeiffer T., Khennouf L., Madry C. (2019). Amyloid β oligomers constrict human capillaries in Alzheimer’s disease via signaling to pericytes. Science.

[123] Gosztyla M.L., Brothers H.M., Robinson S.R. (2018). Alzheimer’s Amyloid-β is an antimicrobial peptide: A review of the evidence. J. Alzheimers Dis..

[124] Moir R.D., Lathe R., Tanzi R.E. (2018). The antimicrobial protection hypothesis of Alzheimer’s disease. Alzheimers Dement..

[125] Vélez J.I., Lopera F., Silva C.T., Villegas A., Espinosa L.G., Vidal O.M., Mastronardi C.A., Arcos-Burgos M. (2020). Familial Alzheimer’s Disease and recessive modifiers. Mol. Neurobiol..

[126] Vélez J.I., Lopera F., Sepulveda-Falla D., Patel H.R., Johar A.S., Chuah A., Tobón C., Rivera D., Villegas A., Cai Y. (2016). APOE*E2 allele delays age of onset in PSEN1 E280A Alzheimer’s disease. Mol. Psychiatry.

[127] Arboleda-Velasquez J.F., Lopera F., O’Hare M., Delgado-Tirado S., Marino C., Chmielewska N., Saez-Torres K.L., Amarnani D., Schultz A.P., Sperling R.A. (2019). Resistance to autosomal dominant Alzheimer’s disease in an APOE3 Christchurch homozygote: A case report. Nat. Med..

